# Peroxisome Proliferator–Activated Receptor-α Agonism With Fenofibrate Does Not Suppress Inflammatory Responses to Evoked Endotoxemia

**DOI:** 10.1161/JAHA.112.002923

**Published:** 2012-08-24

**Authors:** Claire K. Mulvey, Jane F. Ferguson, Jennifer Tabita-Martinez, Stephanie Kong, Rhia Y. Shah, Parth N. Patel, Stephen R. Master, M Haris U. Usman, Kathleen J. Propert, Rachana Shah, Nehal N. Mehta, Muredach P. Reilly

**Affiliations:** Cardiovascular Institute, Perelman School of Medicine, University of Pennsylvania, Philadelphia, PA (C.K.M., J.F.F., J.T.-M., S.K., R.Y.S., P.N.P., M.H.U.U., N.N.M., M.P.R.); Department of Pathology and Laboratory Medicine, Perelman School of Medicine, University of Pennsylvania, Philadelphia, PA (S.R.M.); Center for Clinical Epidemiology and Biostatistics, Perelman School of Medicine, University of Pennsylvania, Philadelphia, PA (K.J.P.); Division of Pediatric Endocrinology, Children's Hospital of Philadelphia, PA (R.S.).

**Keywords:** clinical trials, cytokines, endotoxemia, fenofibrate, inflammation

## Abstract

**Background:**

Data conflict with regard to whether peroxisome proliferator–activated receptor-α agonism suppresses inflammation in humans. We hypothesized that in healthy adults peroxisome proliferator–activated receptor-α agonism with fenofibrate would blunt the induced immune responses to endotoxin (lipopolysaccharide [LPS]), an in vivo model for the study of cardiometabolic inflammation.

**Methods and Results:**

In the Fenofibrate and omega-3 Fatty Acid Modulation of Endotoxemia (FFAME) trial, 36 healthy volunteers (mean age 26±7 years, mean body mass index 24±3 kg/m^2^, 44% female, 72% white) were randomized to fenofibrate 145 mg or placebo daily. After 6 to 8 weeks of treatment, subjects underwent a low-dose LPS challenge. Clinical and blood measurements were collected at randomization, before LPS administration, and serially for 24 hours after LPS administration. We examined area under the curve for evoked responses by treatment group. Compared to placebo, but before LPS challenge, fenofibrate reduced total cholesterol and tended to decrease triglycerides, consistent with achieved therapeutic plasma levels of fenofibric acid. In the placebo group, LPS induced a modest inflammatory response with increased cytokines and chemokines (2- to 4-hour post-LPS 8-fold increase in tumor necrosis factor-α, 9-fold increase in interleukin-6, 9-fold increase in interleukin-10, and 10-fold increase in monocyte chemotactic protein-1; all *P*<0.001) and acute-phase reactants (24-hour post-LPS 15-fold increase in serum amyloid A and 9-fold increase in C-reactive protein; both *P*<0.001). Compared to placebo, however, fenofibrate did not significantly attenuate LPS-induced levels of plasma cytokines, chemokines, or acute-phase proteins.

**Conclusions:**

These data suggest a lack of systemic antiinflammatory properties of fenofibrate at clinically relevant dosing in humans.

**Clinical Trial Registration:**

URL: http://clinicaltrials.gov/ct2/show/NCT01048502. Unique identifier: NCT01048502. **(*J Am Heart Assoc*. 2012;1:e002923 doi: 10.1161/JAHA.112.002923.)**

## Introduction

Inflammation plays a central role in the pathogenesis of cardiometabolic disease. Atherosclerosis and insulin resistance are active inflammatory disorders characterized by the infiltration of inflammatory leukocytes and activation of innate and adaptive immunity.^1^ Peroxisome proliferator–activated receptor-α (PPAR-α), a member of the nuclear steroid receptor family, functions as a transcription factor that regulates metabolic homeostasis by controlling the expression of genes involved in lipid metabolism, inflammation, and atherosclerosis.^2^ Fibrates are synthetic PPAR-α agonists that confer putative benefits on cardiovascular disease risk by improving plasma triglycerides (TGs), high-density lipoprotein (HDL), and the overall atherogenic lipid profile.^3^ In addition, fibrates may modulate inflammatory signaling.^2^ Nevertheless, data conflict as to whether PPAR-α agonism with fibrates produces clinically relevant modulation of inflammatory pathways in humans.

PPAR-α activation is suggested to have a direct inhibitory effect on inflammation through repression of the proinflammatory transcription factors nuclear factor-κB and activator protein-1.^4^ Mice lacking *Ppar-α* have a prolonged response to inflammatory stimuli.^5^ Furthermore, aortas from mice deficient in *Ppar-α* have an exaggerated inflammatory response to in vivo stimulation with endotoxin (lipopolysaccharide [LPS]), as demonstrated by increased interleukin (IL)-6 secretion.^4^ PPAR-α activation with fibrates inhibits the hepatic acute-phase response in vivo in mice^6^ and has been shown to inhibit in vitro production of IL-6 and cell adhesion molecules in human endothelial cells, smooth muscle cells, and macrophages.^7–9^ Despite this experimental evidence, there is limited data to support antiinflammatory effects of fibrates in humans in vivo.^8,10–12^

Experimental endotoxemia has emerged as a controlled model for the study of complex disease inflammatory responses and their modulation in vivo.^13^ Administration of an intravenous bolus of purified *Escherichia coli* endotoxin activates Toll-like receptor-4 signaling and stimulates innate immunity in humans. Remarkably, the short-lived inflammatory responses to endotoxemia induce metabolic perturbations that resemble those observed in insulin resistance, type 2 diabetes, and atherosclerosis.^14–18^ Because experimental endotoxemia studies evoke inflammatory–metabolic responses in otherwise healthy humans, they have the advantage of requiring modest sample sizes to demonstrate directional and causal modulation of the evoked responses.

Here, we utilize experimental endotoxemia to evaluate the antiinflammatory effects of fenofibrate in a randomized, controlled clinical trial in healthy volunteers. We selected a low dose of endotoxin (0.6 ng/kg LPS) to generate a mild inflammatory response that approximates the chronic inflammation of cardiometabolic disease. We demonstrate that fenofibrate treatment for 6 to 8 weeks does not attenuate inflammatory responses during low-dose endotoxemia in humans.

## Methods

### Clinical Trial Design

#### Subjects

Healthy volunteers 18 to 45 years of age with a body mass index between 18 and 30 kg/m^2^ were recruited from the general population of the Delaware Valley. Exclusions included past medical history of inflammatory diseases, pregnancy, use of medication or supplement, or substance use. Physical examination, routine laboratory tests, and electrocardiogram were normal in participants. All subjects were nonsmokers and had no history or signs of arterial hypertension, major disorders in lipid metabolism, or other cardiovascular risk factors. The trial was conducted with the approval of the University of Pennsylvania Institutional Review Board. All participants provided written informed consent. The trial was approved by the US Food and Drug Administration and registered on clinicaltrials.gov as NCT01048502.

#### Study Design

**Figure 1.**
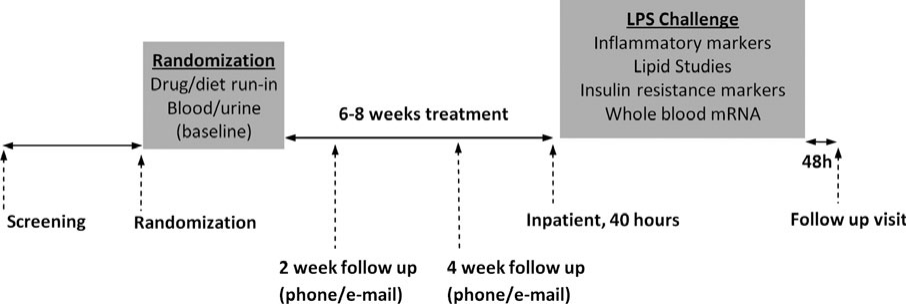
Design of the FFAME study.

An overview of the design of the Fenofibrate and omega-3 Fatty Acid Modulation of Endotoxemia (FFAME) trial is provided in Figure 1. This was an investigator-initiated, double-blind, parallel-group, placebo-controlled study. Participants were randomized to 1 of 4 treatment arms: placebo, fenofibrate (Tricor, Abbott Laboratories) 145 mg/d, or omega-3-acid ethyl ester (Lovaza, GlaxoSmithKline; 465/375 mg EPA/DHA) supplemented at either 900 or 3600 mg/d. The trial was designed a priori to enroll 80 subjects to completion of the inpatient endotoxin protocol across all study arms, with ∼20 subjects per group. This report focuses on the fenofibrate-versus-placebo aspect of the trial, which was prespecified in the trial protocol as a separate hypothesis distinct from omega-3 supplementation. On the basis of results from a comparable previous study, the sample size was chosen a priori to have statistical power of 0.8, at an α level of 0.05, to detect a 27% reduction in the plasma tumor necrosis factor-α (TNF-α) delta area under the curve (ΔAUC) response to endotoxin for the fenofibrate treatment group compared to placebo.^19^

#### Therapeutic Interventions

Tricor tablets were acquired from Abbott Laboratories (North Chicago, IL), and matching placebo was generated by the Investigational Drug Service of Penn's Clinical and Translational Research Center. Lovaza capsules and matching placebos were provided by GlaxoSmithKline (Research Triangle Park, NC). Subjects assigned to Lovaza also were given matching Tricor placebo, whereas subjects in the Tricor group took matching Lovaza placebo. Placebo subjects were assigned both Tricor and Lovaza placebos.

#### Endotoxemia Study Protocol

Subjects were recruited for 3 study visits: visit 1, for screening; visit 2, after a 12-hour overnight fast for randomization and collection of baseline laboratory samples; and visit 3, 6 to 8 weeks after randomization (median treatment duration 41 days), for a 40-hour inpatient stay consisting of an overnight fasting acclimatization phase and a post-LPS study phase. Whole-blood samples for separation of plasma and serum were collected before and serially for 24 hours after intravenous bolus infusion of 0.6 ng/kg US standard reference endotoxin (lot No. CC-RE-LOT-1+2; Clinical Center, Pharmacy Department at the National Institutes of Health, Bethesda, MD). Vital signs were monitored and subjective symptoms recorded for the duration of the inpatient visit.

### Laboratory Methods

Plasma concentrations of fenofibric acid were measured with a validated liquid chromatography–tandem mass spectrometry method (assay range 0.25 to 25 μg/mL) (Frontage Laboratories; Malvern, PA) as described previously.^20^ After ultracentrifugation, plasma total cholesterol, low-density lipoprotein, HDL, TGs, apolipoprotein B, and apolipoprotein A-I were measured enzymatically on a Hitachi 912 Analyzer (Roche Diagnostics; Indianapolis, IN) in a Centers for Disease Control–certified lipid laboratory. Serum amyloid A and high-sensitivity C-reactive protein (CRP) were measured by latex particle–enhanced immunonephelometry on a Behring Nephelometer II Analyzer (Siemens Diagnostics; Munich, Germany). Plasma levels of TNF-α, IL-6, IL-10, and monocyte chemotactic protein-1 (MCP-1) were measured with sandwich ELISAs according to the manufacturer's instructions (Quantikine, R&D Systems; Minneapolis, MN). The intra- and interassay coefficients of variation were 7.5% and 14.8% for TNF-α, 5.8% and 14.3% for IL-6, 7.0% and 8.0% for IL-10, and 7.9% and 11.1% for MCP-1. The lower limits of quantification were 0.4 pg/mL for TNF-α, 0.154 pg/mL for IL-6, 0.78 pg/mL for IL-10, and 31.2 pg/mL for MCP-1.

### Statistical Analysis

Efficacy analyses included, a priori, data from all 36 participants who completed the inpatient endotoxin challenge. Unless otherwise specified, data are reported as means±standard deviations for continuous variables and as proportions for categorical variables. Continuous variables with non-normal distributions were log-transformed for modeling where indicated. Continuous variables were compared by treatment group with Student *t* tests or the nonparametric Mann-Whitney *U* test in cases in which homogeneity of variance was violated by Levene test. For discrete variables, group differences were assessed using the Fisher exact test. Peak responses after LPS were compared to baseline with Wilcoxon signed-rank tests. To evaluate endotoxin effects over time, AUC was calculated for outcome variables by using the trapezoidal rule. The area representing the pre-LPS baseline was then subtracted out for an incremental, or ΔAUC, which was compared by treatment group. The primary outcome for analysis was the ΔAUC, but we also examined peak response and total AUC for evoked responses. A *P* value <0.05 was considered to indicate statistical significance. We did not correct for multiple testing; plasma TNF-α and IL-6 were primary endpoints, with additional traits analyzed to provide complementary information about the impact on diverse inflammatory pathways. Statistical analyses were performed in Stata 12.0 software (Stata Corporation; College Station, TX).

## Results

### Baseline Characteristics of Participants Do Not Differ by Treatment Group

**Figure 2.**
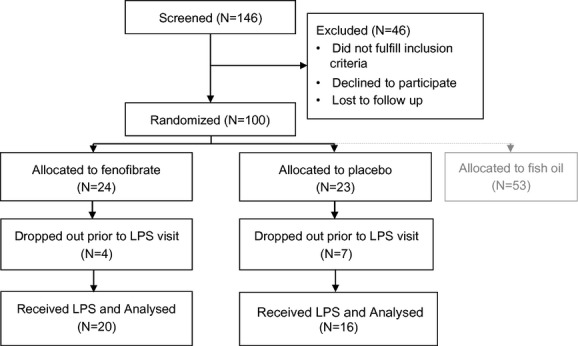
Flow diagram for the FFAME study. The fenofibrate and placebo arms of the trial were a portion of a larger, multi-arm trial.

Forty-seven participants (24 randomized to fenofibrate and 23 to placebo) were enrolled in the FFAME trial (Figure 2). There were 11 dropouts before the inpatient endotoxin visit, with 36 subjects completing the full experimental protocol. Of the fenofibrate group, 1 subject was discontinued by the investigator shortly after randomization because of a new diagnosis of systemic lupus erythematosus, 1 withdrew because of schedule conflicts with the remaining visits, and 2 subjects were lost to follow-up before the endotoxin visit. Of the placebo group, 3 subjects were discontinued before the LPS visit because of noncompliance with study medication as prespecified in the protocol (found to have taken <80% of study medication at the preadmission pill count), 1 subject had an acute seasonal allergy that required antiallergy medication, 1 subject withdrew because the subject no longer wished to participate, and 1 subject withdrew because of schedule conflicts.

As specified a priori in the protocol, only subjects who received LPS at the inpatient visit (n=36) were included in the efficacy analysis of fenofibrate effects on induced inflammation. Completing participants were healthy, and the 2 groups were well balanced at baseline (Table 1). Anthropometric measures of adiposity, blood pressure, inflammatory markers, and plasma lipoproteins were within normal limits for both treatment groups.

**Table 1.** Baseline Characteristics of FFAME Study Participants

Variable	Placebo (n=16)	Fenofibrate (n=20)	P*
Age, y	27.2±1.8	25.3±1.4	0.41
Female, n (%)	6 (37.5)	10 (50)	0.51†
Race, n (%)			
White	12 (75.0)	14 (70.0)	0.55†
Black	2 (12.5)	5 (25.0)	
Asian	2 (12.5)	1 (5)	
Body mass index, kg/m^2^	23.7±3.4	23.8±3.3	0.94
Waist circumference, cm	83.9±8.8	78.8±10.8	0.14
Systolic blood pressure, mm Hg	120.4±12.8	125.4±11.1	0.22
Diastolic blood pressure, mm Hg	74.3±8.1	75.0±9.2	0.82
Heart rate, bpm	65.3±9.5	64.7±7.9	0.83
TNF-α, pg/mL	1.3±0.8	1.2±0.5	0.60
IL-6, pg/mL	1.1±0.5	1.2±0.5	0.89
IL-10, n detectable (%)	2 (13)	7 (35)	0.25‡
MCP-1, pg/mL	146.5±36.3	144.1±49.6	0.87
High-sensitivity CRP, mg/L	0.80±1.39	1.20±1.56	0.44
Serum amyloid A, mg/L	4.2±3.4	4.2±3.9	0.97
Apolipoprotein A-I, mg/dL	149.6±36.8	139.0±38.9	0.41
Apolipoprotein B, mg/dL	76.7±30.4	74.5±21.8	0.80
Total cholesterol, mg/dL	177.4±39.4	170.3±34.5	0.57
LDL cholesterol, mg/dL	97.6±39.3	96.0±28.7	0.89
HDL cholesterol, mg/dL	61.4±18.2	58.7±17.0	0.65
TGs, mg/dL	91.6 (46.1)	78.0±39.8	0.34§§
Phospholipids, mg/dL	206.1±45.3	192.6±34.5	0.32

Values are n (%) or mean±standard deviation. CRP indicates C-reactive protein; LDL,low-density lipoprotein; HDL, high-density lipoprotein; and TGs, plasma triglycerides.

* *P* values obtained from 2-sided Student *t* test unless otherwise indicated.

† *P* value is from Fisher exact test.

‡ Given the large proportion of individuals below the limit of detection, data are presented as the n (%) of individuals above the limit of detection. *P* value obtained from Fisher exact test.

§§ Raw data are presented, but log-transformed values were used in analysis.

### Subjects Adhere to Fenofibrate Treatment With Minimal Adverse Effects

Exposure to fenofibrate was confirmed through pill count (<20% of study medication remaining at preadmission count) and the measurement (batched) of plasma fenofibric acid drug levels in samples collected the morning of admission for the inpatient visit 1 day before LPS administration. None of the placebo subjects (n=16) had detectable plasma levels of fenofibric acid at preadmission. All but 1 of the fenofibrate subjects had detectable levels (n=19; mean concentration 13.3±6.3 μg/mL), consistent with peak steady-state levels achieved in published pharmacokinetic trials of fenofibrate 145 mg (12.8 μg/mL)^21^ and the range seen with other bioequivalent formulations of fenofibrate (9.6 to 12.3 μg/mL).^22–23^ The fenofibrate subject without detectable levels was included in all analyses on the basis of intention to treat, but removal did not alter interpretation of the results.

Adverse events that occurred in the fenofibrate and placebo arms of the FFAME trial are summarized in Table 2. In general, study medication and procedures were well tolerated. No serious adverse events were reported.

**Table 2.** Summary of Adverse Events by Treatment Group

Events	Fenofibrate (n=24)	Placebo (n=23)
Hemoglobin drop >2 g, n	1	1
Temperature >101°F, n	0	2
Myalgias, n	0	1
Nausea or emesis, n	1	1
Diarrhea, n	1	1
Dyspepsia, n	2	2
Dry mouth, n	0	2
Pharyngitis, n	1	1
Diagnosis of lupus, n	1	0
Cough, flu-like symptoms, n	4	4
Headache, n	1	0
Dizziness, n	2	1
Lethargy, n	0	1
Pruritus, n	0	1
Urinary tract infection, n	0	1
Total, n	14	19
% Total	42	58

### Fenofibrate Modulates Lipids but Has Little Effect on Inflammatory Markers Before LPS

Changes in plasma lipids after treatment but before LPS administration are presented in Table 3. As expected, compared to placebo, fenofibrate reduced total cholesterol (*P*=0.009), low-density lipoprotein (*P*=0.001), and apolipoprotein B (*P*=0.008). Fenofibrate also tended to decrease TGs compared to placebo before LPS but did not reach statistical significance (*P*=0.41). This modest trend in TGs is consistent with the relatively low TGs at baseline and published effects of fenofibrate in such settings.^24^ In contrast, 6 to 8 weeks of fenofibrate had no effect on HDL, phospholipids, or apolipoprotein A-I compared to placebo before endotoxemia.

**Table 3.** Changes in Lipid Parameters After 6 to 8 Weeks of Treatment but Before Endotoxin

	Randomization	Before LPS	Absolute Δ	P*
Total cholesterol, mg/dL				
Fenofibrate	170.3±34.5	136.9±30.8	−33.4±18.2	0.0094
Placebo	177.4±39.4	160.9±35.0	−16.4±18.6	
LDL cholesterol, mg/dL				
Fenofibrate	96.0±28.7	72.6±28.4	−23.4±14.2	0.0014
Placebo	97.6±39.3	92.3±32.4	−5.4±16.9	
TGs, mg/dL				
Fenofibrate	78.0±39.8	57.0±22.5	−21.0±38.9	0.41†
Placebo	91.6±46.1	76.7±32.9	−14.9±31.7	
HDL cholesterol, mg/dL				
Fenofibrate	58.7±17.0	52.9±13.4	−5.8±9.1	0.50
Placebo	61.4±18.2	53.3±15.6	−8.1±10.7	
Apolipoprotein A-I, mg/dL				
Fenofibrate	139.0±38.9	117.8±23.5	−21.1±22.1	0.64
Placebo	149.6±36.8	125.2±33.5	−24.4±19.6	
Apolipoprotein B, mg/dL				
Fenofibrate	74.4±21.8	55.1±19.2	−19.3±13.2	0.0079
Placebo	76.7±30.4	68.8±23.5	−7.9±10.5	
Phospholipids, mg/dL				
Fenofibrate	192.6±34.5	165.6±26.6	−27.0±24.0	0.61
Placebo	206.1±45.3	183.2±33.9	−22.9±23.7	

Values are given as mean±standard deviation. LPS indicates lipopolysaccharide; LDL, low-density lipoprotein; TGs, plasma triglycerides; and HDL, high-density lipoprotein.

* *P* values obtained from 2-sided Student *t* test comparing absolute change from baseline to before LPS administration by treatment group.

† Raw data are presented, but log-transformed data were used in analysis.

Changes in inflammatory parameters before endotoxin are presented in Table 4. Compared to placebo, fenofibrate did not modulate plasma levels of TNF-α (*P*>0.99) or MCP-1 (*P*=0.66) but appeared to reduce IL-6 levels (*P*=0.003); however, the difference between the fenofibrate and the placebo groups in the effect on IL-6 was driven by an increase in the placebo group. At baseline and after treatment, plasma levels of IL-10 were below the lower limit of detection in the majority of subjects in both study groups (65% of fenofibrate versus 87% of placebo subjects at baseline, *P*=0.25; 85% of fenofibrate versus 69% of placebo subjects after treatment, *P*=0.42).

**Table 4.** Changes in Inflammatory Parameters After 6 to 8 Weeks of Treatment but Before Endotoxin

	Randomization	Before LPS	Absolute Δ	P*
TNF-α, pg/mL				
Fenofibrate	1.2±0.5	1.1±0.4	−0.1±0.3	>0.99†
Placebo	1.3±0.8	1.5±1.2	0.2±1.1	
IL-6, pg/mL				
Fenofibrate	1.1±0.5	2.0±0.7	0.9±0.7	0.0028†
Placebo	1.2±0.5	3.6±2.0	2.4±1.7	
MCP, pg/mL				
Fenofibrate	144.1±49.6	140.2±33.6	−3.8±42.2	0.66†
Placebo	146.5±36.3	147.4±33.6	0.9±21.5	
CRP, mg/L				
Fenofibrate	1.2±1.6	0.8±1.0	−0.4±1.5	0.36
Placebo	0.8±1.4	0.8±0.9	−0.02±1.1	
Serum amyloid A, mg/L				
Fenofibrate	4.2±3.9	3.1±0.8	−1.1±3.9	0.66†
Placebo	4.2±3.4	3.7±2.1	−0.4±1.6	

Values are given as mean±standard deviation. LPS indicates lipopolysaccharide; MCP, monocyte chemotactic protein; and CRP, C-reactive protein.

* *P* values obtained from 2-sided Student *t* test comparing absolute change from baseline to pre-LPS by treatment group.

† Violated assumptions of homogeneity of variances using Levene test; *P* value obtained from Mann-Whitney *U* nonparametric test.

### Fenofibrate Does Not Modulate Clinical Responses to Evoked Endotoxemia

**Figure 3.**
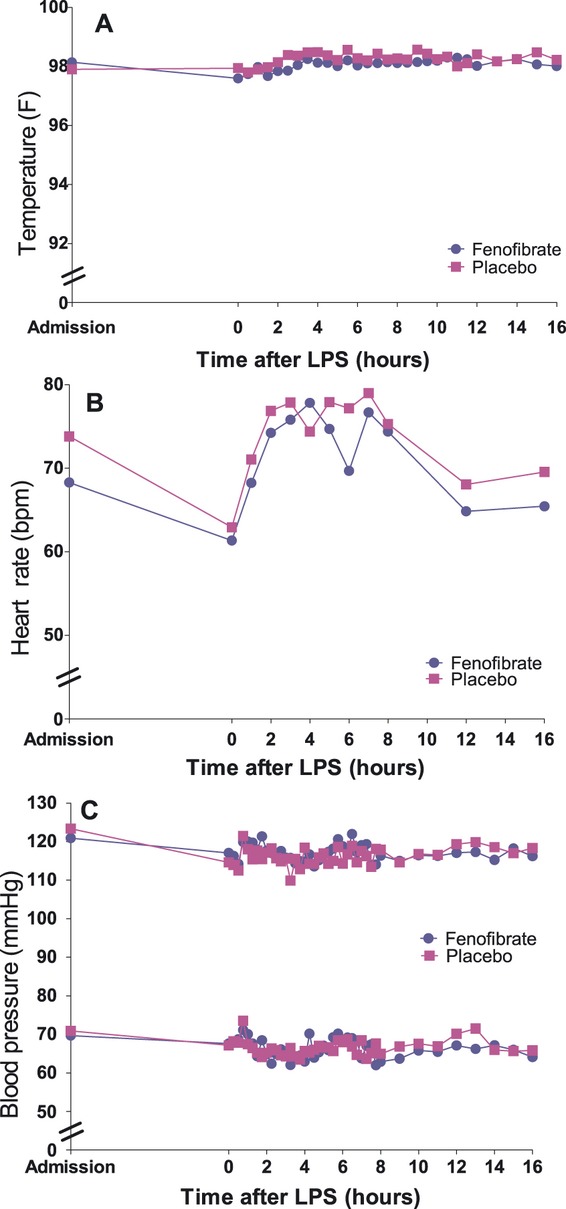
Fenofibrate has no effect on the clinical responses to endotoxemia. Temperature increased slightly (A) and heart rate increased modestly (B) in participants after LPS administration, whereas systolic and diastolic blood pressure did not change significantly (C). Mean values at each time point are presented by treatment group. Standard deviations are not presented because of marked overlap between groups. No significant differences in peak or ΔAUC values were seen by treatment group for any clinical parameter. LPS indicates lipopolysaccharide.

As expected, low-dose endotoxemia produced a mild, transient clinical response (Figure 3), characterized by peak increases in temperature (placebo +0.6°F and fenofibrate +0.7°F, *P*=0.001 for before LPS versus after LPS for both groups) and heart rate (placebo +16.1 beats per minute, *P*<0.001 for before LPS versus after LPS; fenofibrate +17.3 beats per minute, *P*=0.002 for before LPS versus after LPS). Overall, post-LPS decreases in systolic blood pressure (placebo −4.8 mm Hg, *P*=0.074; fenofibrate −3.4 mm Hg, *P*=0.74) and diastolic blood pressure (placebo −3.7 mm Hg, *P*=0.24; fenofibrate −4.8 mm Hg, *P*=0.091) were slight and not statistically significant compared to pre-LPS values. The temperature (Figure 3A), heart rate (Figure 3B), systolic blood pressure (Figure 3C), and diastolic blood pressure (Figure 3C) responses to endotoxemia did not differ with fenofibrate treatment compared to placebo (ΔAUC: *P*=0.27, *P*=0.70, *P*=0.34, and *P*=0.63, respectively).

### Fenofibrate Fails to Suppress the Inflammatory Response to Evoked Endotoxemia

**Figure 4.**
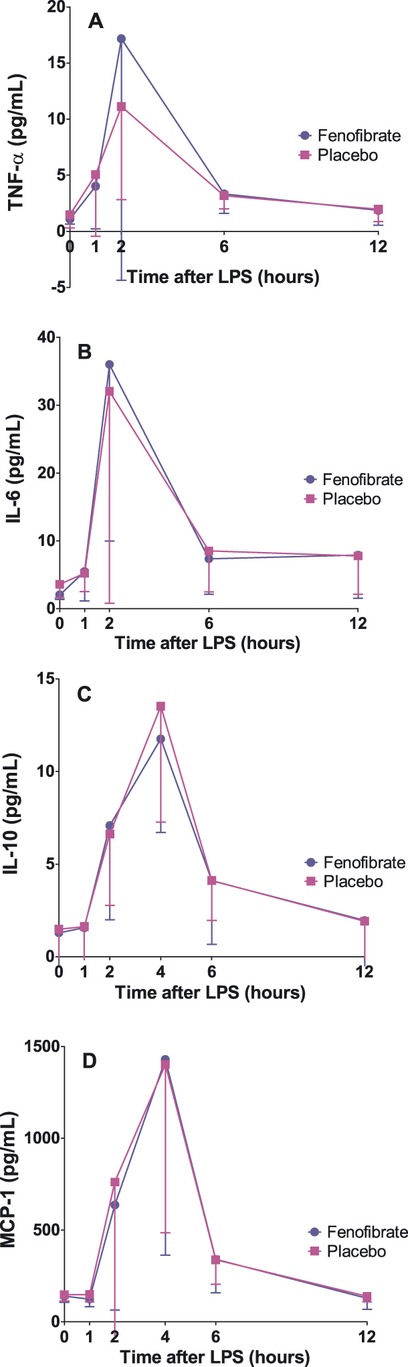
Fenofibrate does not suppress the cytokine and chemokine response to endotoxemia. Changes in cytokine and chemokine parameters after endotoxin administration are presented as means with error bars indicating standard deviations. For clarity of presentation, 1-sided error bars are shown. Fenofibrate did not significantly modulate the cytokine or chemokine response to endotoxemia when analyzed as ΔAUC, total AUC, or peak response. LPS indicates lipopolysaccharide.

As expected, LPS induced a robust inflammatory response. Plasma levels of the proinflammatory cytokines TNF-α (Figure 4A) and IL-6 (Figure 4B) increased significantly after LPS administration compared to before LPS administration, peaking at 2 hours (placebo group, TNF-α peak after LPS 11.2±8.2 pg/mL, 8-fold increase, *P*<0.001; fenofibrate group, TNF-α peak after LPS 17.2±21.5 pg/mL, 16-fold increase, *P*<0.001; placebo group, IL-6 peak after LPS 32.6±30.7 pg/mL, 9-fold increase, *P*<0.001; fenofibrate group, IL-6 peak after LPS 36.9±25.2 pg/mL, 18-fold increase, *P*<0.001). The response of either cytokine did not, however, differ significantly by treatment (ΔAUC: TNF-α, *P*=0.43; IL-6, *P*=0.13). IL-10 (Figure 4C) also increased significantly after LPS, peaking at 4 hours (placebo group, peak 13.7±6.3 pg/mL, 9-fold increase, *P*<0.001; fenofibrate group, peak 12.0±5.3 pg/mL, 9-fold increase, *P*<0.001); however, the IL-10 response to LPS also did not differ by treatment (ΔAUC, *P*=0.66). Similarly, although the chemokine MCP-1 (Figure 4D) peaked 4 hours after LPS administration (placebo group, peak 1460±979 pg/mL, 10-fold increase, *P*<0.001; fenofibrate group, peak 1440±1060 pg/mL, 10-fold increase, *P*<0.001), its response to LPS was not suppressed by fenofibrate (ΔAUC, *P*=0.93).

**Figure 5.**
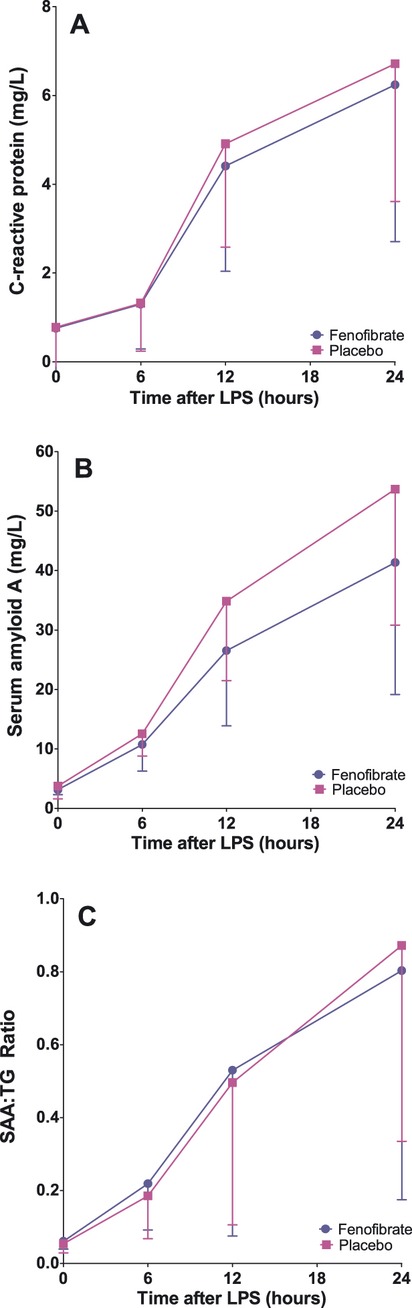
Fenofibrate does not modulate hepatic acute-phase responses to endotoxemia. High-sensitivity CRP (A) and serum amyloid A (SAA) (B) increased after endotoxin. CRP responses did not differ significantly by treatment group. SAA trended lower in the fenofibrate group but did not reach statistical significance (ΔAUC,* P*=0.12), and the SAA:TG ratio (C) did not differ by treatment (ΔAUC,* P*=0.68). Values shown are mean±standard deviations. LPS indicates lipopolysaccharide.

**Figure 6.**
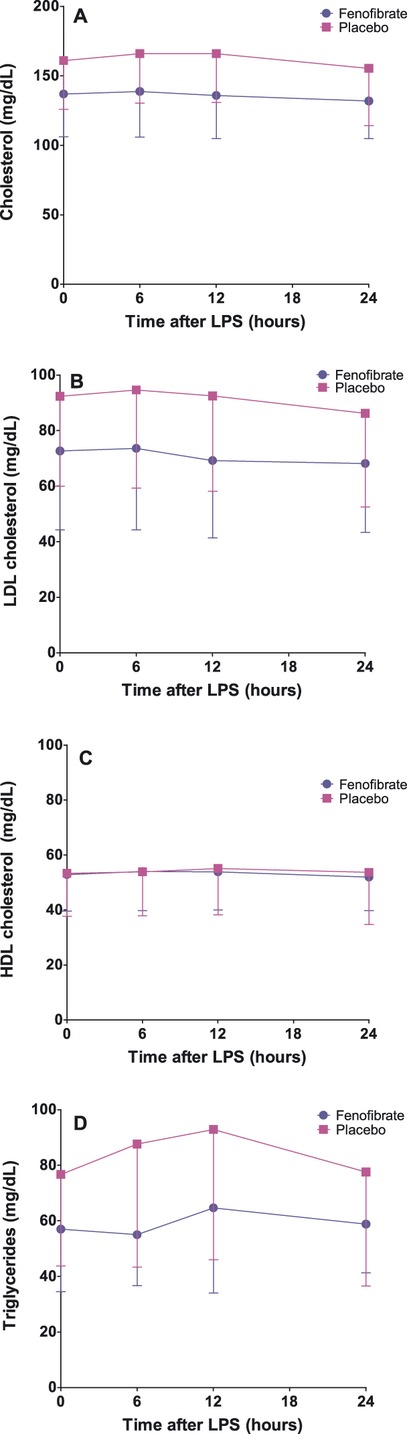
Lipid responses to endotoxemia did not differ by treatment group. Total cholesterol (A), LDL cholesterol (B), HDL cholesterol (C), and triglyceride (D) responses after endotoxin administration are presented as mean±standard deviation. No significant differences were observed by treatment group for any lipid variable as measured by ΔAUC. LPS indicates lipopolysaccharide; LDL, low-density lipoprotein; and HDL, high-density lipoprotein.

Hepatic acute-phase reactants also increased in both treatment groups after LPS (Figure 5), peaking at 24 hours (CRP, 9-fold increase for the placebo group and 8-fold for the fenofibrate group; serum amyloid A, 15-fold increase for the placebo group and 13-fold for the fenofibrate group; all *P*<0.001 compared to before LPS). Fenofibrate did not suppress CRP levels compared with placebo (Figure 5A) (ΔAUC, *P*=0.61). The serum amyloid A response to LPS trended lower with fenofibrate treatment (Figure 5B) (ΔAUC, *P*=0.12). However, when serum amyloid A levels were corrected for TG levels (Figure 5C), there was no longer any trend toward lower levels with fenofibrate treatment (ΔAUC, *P*=0.68). Finally, compared to placebo, fenofibrate did not modulate lipoprotein responses to endotoxemia (Figure 6).

## Discussion

PPAR-α agonism with fibrates is proposed to reduce atherogenic inflammation. We used a low-dose endotoxemia protocol to assess the antiinflammatory effects of fenofibrate, at the maximum dose prescribed for lipid abnormalities, on an induced inflammatory state in healthy humans. Fenofibrate lowered lipoproteins as expected before LPS. Despite direct evidence (via measurement of fenofibric acid levels in plasma) that treated individuals were exposed to therapeutic drug levels before endotoxin administration,^21^ fenofibrate failed to significantly attenuate the clinical, innate immune, or acute-phase responses to endotoxemia in vivo. These findings suggest limited, if any, systemic antiinflammatory properties of fenofibrate in healthy humans at clinically relevant dosing.

Given the effects of PPAR-α on lipid homeostasis, fibrates have been used clinically for cardiovascular risk reduction, especially in high-risk patients with type 2 diabetes mellitus. Initial clinical trials with PPAR-α agonists, such as the Helsinki Heart Study^25^ and the Veterans Affairs HDL Intervention Trial,^26^ demonstrated a significant reduction in major cardiovascular events compared to placebo. In the Fenofibrate Intervention and Event Lowering in Diabetes (FIELD) trial in subjects with type 2 diabetes, fenofibrate did not have a significant effect on the primary composite endpoint of nonfatal myocardial infarction and coronary heart disease death but did seem to reduce the incidence of nonfatal myocardial infarction.^27^ In the Action to Control Cardiovascular Risk in Diabetes (ACCORD) trial, fenofibrate treatment in combination with simvastatin failed to produce a reduction in major cardiovascular events beyond that seen with simvastatin alone, although post hoc analyses suggest potential benefit in the subgroup of patients with the most elevated baseline TGs and lowest HDL.^28^ Thus, the FIELD trial, in which concomitant statin use was prevalent, and the ACCORD trial, in which all were treated with statins, have called into question the utility of fibrates in the current era of widespread statin use. Controversy exists about whether fibrates' cardiovascular benefits might be present only in specific at-risk populations, particularly those with insulin resistance and related lipid abnormalities. Furthermore, whether fibrates confer any antiinflammatory benefit and whether such benefit might be observed in chronic insulin-resistant inflammatory states remains unknown.

Previous studies suggest that PPAR-α agonism inhibits the expression of mediators that promote inflammation within atherosclerotic plaques. Mice lacking the *Ppar-α* gene have a prolonged response to topical inflammatory stimuli compared to wild-type mice.^5^ Long-term treatment with fenofibrate blocked the IL-6–induced acute-phase response in wild-type but not in *Ppar-α*–deficient mice, which suggests a role for PPAR-α as a modulator of the immune response at the hepatic level.^6^ Pharmacological PPAR-α agonism reduced TNF-α levels in murine in vivo LPS models of acute lung injury^29^ and neuroinflammation.^30^ Fenofibrate also prevented the IL-1–induced secretion of IL-6 in a dose-dependent manner in human aortic smooth muscle cells^4^ and inhibited the TNF-α–mediated production of vascular cell adhesion molecule-1 in human endothelial cells.^7^ However, conflicting evidence suggests that PPAR-α actually may have proinflammatory in vivo effects. In a mouse model of endotoxemia, mice treated with PPAR-α agonists before in vivo LPS challenge had 5 times higher plasma TNF-α levels than vehicle-treated animals.^31^ The relevance of these mouse and cell data to fibrate actions in humans in vivo remains unclear.

In human clinical trials, there is limited evidence for the antiinflammatory properties of fibrates. Two small studies of patients mostly with established atherosclerosis found significant reductions in CRP and cytokines with 4 weeks of fenofibrate treatment, but these studies lacked a placebo control.^8,10^ A larger 3-month trial in subjects with mixed hyperlipidemia did report significant reductions in CRP with fenofibrate compared to placebo.^11^ In men with abdominal adiposity and the metabolic syndrome, 6 months of gemfibrozil treatment also decreased CRP but failed to reduce plasma IL-6 or TNF-α levels.^12^ Most trials of fibrates, however, did not address their antiinflammatory effects.

In this context, we sought to test the hypothesis that fenofibrate would blunt inflammatory responses during low-grade endotoxemia in healthy humans. This model is of proven relevance to cardiometabolic disease and provides a probe for the study of therapeutic and genomic influences on inflammatory effects in these disorders.^17, 32–33^ Abundant evidence links Toll-like receptor-4 signaling and subsequent activation of innate immunity with the pathogenesis of insulin resistance and atherosclerosis. We and others have shown that experimental endotoxemia induces adipose inflammation, insulin resistance, and HDL dysfunction in humans.^14–18^ Furthermore, endogenous ligands generated in obesity and atherosclerosis, such as fatty acids and modified lipids, can promote Toll-like receptor-4 signaling.^34^ In fact, deletion of Toll-like receptor-4 attenuated diet-induced obesity, insulin resistance,^35^ and atherosclerosis^36^ in rodent models. Importantly, endotoxemia protocols have been used safely in humans for decades to test the efficacy of numerous antiinflammatory compounds.^37–40^

To our knowledge, the present study is the first human study to evaluate the antiinflammatory effects of fenofibrate in healthy subjects submitted to evoked inflammation. Our study design has the advantage of interrogating induced inflammatory responses over time, which may provide greater insight into immune-modulatory interventions than that derived from single–time-point estimates in population studies or trials. Because our study was conducted in healthy volunteers without medical comorbidities, we minimized heterogeneity between subjects at baseline. The model permits direct assessment of interventions on the directional impact of induced inflammation, avoiding confounding and reverse causation, which are features of observational studies in which inflammatory changes may result from risk factors and disease rather than being causal.

Our study design has some limitations. As an acute model, experimental endotoxemia is not equivalent to the chronic inflammation of cardiometabolic disease. It remains possible that fenofibrate suppresses other proinflammatory mediators that were not measured in this study. However, we did examine multiple domains of inflammation and specifically selected biomarkers of known relevance to cardiometabolic disease risk. There was a 23% dropout rate before the inpatient endotoxin visit. This was anticipated, however, and the study was designed and powered on the basis of projected LPS visit completion, which reflected the rate that actually was observed. Of note, subjects who dropped out did not differ obviously from completers in demographics or clinical parameters.

Our results indicate that fenofibrate treatment, at dosages commonly used for lipid abnormalities and heart disease, does not suppress the clinical or inflammatory responses to low-dose endotoxin in healthy humans. These results suggest that the systemic antiinflammatory properties of fenofibrate are limited.
